# Taming Pancreatic Cancer: *Ardisia virens* Kurz-Derived 4-Hydroxy-2-Methoxy-6-Tridecylphenyl Acetate as a Potent Tubulin Polymerization Inhibitor for Targeted Pancreatic Ductal Adenocarcinoma Therapy

**DOI:** 10.7150/ijms.104112

**Published:** 2025-01-13

**Authors:** Chia-Hung Yen, Chien-Ju Lin, Peng-Yu Chen, Yi-Jin Chen, Ling-Rung Wei, Pei-Hsuan Chen, Yi-Chen Yeh, Lily Hui-Ching Wang, Hsun-Shuo Chang, Wan-Chi Tsai

**Affiliations:** 1 Graduate Institute of Natural Products, College of Pharmacy, Kaohsiung Medical University, Kaohsiung 80708, Taiwan.; 2 Drug Development and Value Creation Research Center, Kaohsiung Medical University, Kaohsiung 80708, Taiwan.; 3 School of Pharmacy, College of Pharmacy, Kaohsiung Medical University, Kaohsiung 80708, Taiwan.; 4 Department of Medical Laboratory Science and Biotechnology, College of Health Sciences, Kaohsiung Medical University, Kaohsiung 80708, Taiwan.; 5 Department of Pathology and Laboratory Medicine, Kaohsiung Veterans General Hospital, Kaohsiung 81362, Taiwan.; 6 Department of Biological Sciences, College of Science, National Sun Yat-sen University, Kaohsiung 80424, Taiwan.; 7 School of Medicine, National Tsing Hua University, Hsinchu 300, Taiwan.; 8 Institute of Molecular and Cellular Biology, National Tsing Hua University, Hsinchu 300, Taiwan.; 9 Department of Laboratory Medicine, Kaohsiung Medical University Hospital, Kaohsiung 807, Taiwan.

**Keywords:** pancreatic ductal adenocarcinoma, 4-hydroxy-2-methoxy-6-tridecylphenyl acetate, *Ardisia virens* Kurz

## Abstract

Pancreatic ductal adenocarcinoma (PDAC) is a major global health challenge owing to late diagnosis and inherently metastatic nature. Although surgical intervention offers a potential remedy, only few patients are eligible, and drug resistance further complicates treatment. The therapeutic limitations have catalyzed a search for alternative treatments, particularly natural products. High-throughput screening identified six extracts from the Ardisia genus, with four from Ardisia virens Kurz, and 4-hydroxy-2-methoxy-6-tridecylphenyl acetate (HMTA) as the most potent candidate. Herein, we explored the anti-cancer effects of HMTA on PDAC and found it induced strong cytotoxic effects on BxPC-3 and PANC-1 pancreatic cancer cell lines. HMTA inhibited cell proliferation and induced apoptosis, as evidenced by annexin V/PI labeling and caspase 3 activation. HMTA halted cancer cell proliferation at the G2/M phase and induced multinucleation. Molecular docking analysis revealed that HMTA potentially could interact with tubulin, and *in vitro* assay confirmed it suppresses tubulin polymerization. HMTA significantly inhibited BxPC-3 xenograft tumor growth in mice. Overall, these findings suggested that HMTA is a promising candidate for PDAC therapy.

## Introduction

Pancreatic ductal adenocarcinoma (PDAC) is a major cause of cancer-related deaths worldwide. Its mortality rate closely matches its incidence because it is typically diagnosed at a late stage and has an inherently metastatic nature 1, 2. Despite a modest improvement in prognosis over the past twenty years 3, only surgery offers a potential cure; however, Unfortunately, <20% percent of patients are eligible owing to late-stage diagnosis, resulting in a 10% five-year survival rate 4. Introduced in 1997, gemcitabine became a primary treatment for pancreatic cancer; however, resistance to it has reduced its efficacy 5. In 2011, the FOLFIRINOX regimen, which combined several agents demonstrated better outcomes than gemcitabine alone; however, with significant toxicity 2, 6. This challenging therapeutic landscape highlights the need to explore alternative treatments for pancreatic cancer, including bioactive compounds from natural products.

Natural products have long been sources of medicinal agents for many centuries, owing to the chemical diversity, biochemical specificity, and various molecular properties, making them ideal candidates for bioactivity screening. We screened over 3,000 methanolic extracts from the library of indigenous plant extracts in Taiwan located at the Natural Product Libraries and High-Throughput Screening Core Lab of Kaohsiung Medical University 7. Of these, 29 extracts showed significant toxicity to pancreatic cancer cells. Notably, six of these extracts were derived from the *Ardisia* plant, with four explicitly originating from various parts of *Ardisia virens* Kurz, including its stem bark, stem, root, and fruit. Multiple compounds extracted from *A. Virens* exhibit strong anticancer properties 8. Notably, 4-hydroxy-2-methoxy-6-tridecylphenyl acetate (HMTA), also referred to as ardisiphenol D, an alkyl phenol derivative, exhibits the highest activity and induces apoptosis in human non-small-cell lung and pancreatic cancer cell lines (A549 and PNAC-1, respectively) 9-11. To address the urgent need for new therapeutic strategies for PDAC, this study explored the mechanisms of HMTA and its *in vivo* anticancer potential for treating pancreatic cancer. Building on previous findings that identified HMTA's cytotoxic effects, this research elucidates its underlying molecular mechanisms, with a specific focus on its interaction with tubulin and ability to inhibit tubulin polymerization. Additionally, this study evaluates HMTA's *in vivo* efficacy against PDAC, with an aim of establishing HMTA as a promising candidate for targeted pancreatic cancer therapy. The broader significance of this study lies in its potential to introduce a novel, plant-derived therapeutic agent that addresses the limitations of current treatments and offers new hope for improving the outcomes of patients with PDAC.

## Methods

### Preparation of 4-hydroxy-2-methoxy-6-tridecylphenyl acetate

Stems of *A. virens* were collected in August 2016, in Meinon, Kaohsiung, Taiwan. A voucher specimen (No. Chen 6105) was in the Herbarium of the College of Pharmacy, Kaohsiung Medical University, Taiwan. HMTA was isolated, and its structure elucidated as described in a previous report 8.

### Cell cultures

Human pancreatic cancer cell lines (BxPC-3 and PANC-1) were purchased from the Bioresource Collection and Research Center in Taiwan. PANC-1 cells were cultured in Dulbecco's modified Eagle's medium (DMEM) containing 10% fetal bovine serum (FBS) and 1% penicillin/streptomycin (P/S). BxPC-3 cells were cultured in RPMI-1640 medium containing 10% FBS, 1% P/S, and 1% sodium pyruvate. HPDE-6/E6E7 cells were cultured routinely in keratinocyte serum-free (KSF) medium supplemented by bovine pituitary extract and epidermal growth factor (Gibco-BRL, Grand Island, NY). All cells were cultured in a 37°C incubator with 5% CO_2_ 12.

## Materials and equipment

The following materials and equipment were used in this study: 3-[4,5-Dimethylthiazol-2-yl]-2,5-diphenyltetrazolium bromide (MTT) solution (Sigma-Aldrich, St. Louis, MO, USA), lactate dehydrogenase (LDH) assay (LDH Cytotoxicity Assay, ScienCell Research Laboratories, Teaneck, NJ, USA), the enzyme linked immunosorbent assay (ELISA) reader (Multiskan EX, Thermo Scientific, Vantaa, Finland), Cell Proliferation ELISA, BrdU (colorimetric) Kit (Roche Applied Science, Indianapolis, IN, USA), Cell Proliferation ELISA, BrdU (colorimetric) Kit (Roche Applied Science, Indianapolis, IN, USA), Dead Cell Apoptosis Kit with Annexin V-Alexa Fluor™ 488 and PI (Invitrogen, Carlsbad, CA, USA), flow cytometer (BD Biosciences, Palo Alto, CA, USA), FITC Active Caspase-3 Apoptosis Kit (BD Biosciences), WinMDI 2.9 software (BD Biosciences), RIPA lysis buffer (Cell Signaling Technology, Beverly, MA, USA), polyvinylidene difluoride (PVDF) membranes (Millipore, Bedford, MA, USA), ECL Western Blotting Detection System (GE Healthcare, Buckinghamshire, UK), Tubulin Polymerization Assay Kit (#BK011P, Cytoskeleton Inc., Denver, CO). Nude mice (BALB/cAnN.Cg-Foxnlnu/CrlNarl) were obtained from BioLASCO Co., Ltd. (Taiwan).

### Cell viability and lactate dehydrogenase assays

To evaluate the cytotoxicity of HMTA, we used the MTT assay following the method outlined by Tsai *et al.* 12. Five thousand cells/well were seeded in 96-well plates and incubated at 37 °C overnight. Thereafter, the cells were treated with varying concentrations of HMTA for 24 h, with Dimethyl sulfoxide (DMSO) serving as the vehicle control. The purple formazan crystals produced were dissolved in DMSO, and absorbance was measured at 595 nm using a microplate reader (Molecular Devices, CA, USA). Cell viability was assessed by comparing the absorbance of the treated samples to that of the control group. Results were presented as the mean ± standard deviation of six independent experiments conducted in triplicate. Additionally, *in vitro* cytotoxic effects of HMTA were evaluated using the LDH assay. Absorbance was read at 450 nm using an ELISA reader.

### Proliferation assay

To evaluate the effect of HMTA on cell proliferation, the BrdU incorporation assay was used as described by Tsai *et al.* 12. Five thousand cells per well were seeded in 96-well plates and treated with varying concentrations of HMTA for 24 h. Thereafter, cells were labeled with BrdU for 4 h at 37 °C using the Cell Proliferation Kit according to the manufacturer's instructions. The cells were subsequently fixed and incubated with an anti-BrdU-peroxidase antibody (diluted 1:100) for 30 min at room temperature. The absorbance of the reaction product was measured at 450 nm using an ELISA reader.

### Annexin V/propidium iodide assay

To investigate HMTA-induced apoptosis, cells were treated with varying concentrations of HMTA or DMSO (control) for 24 h. The Annexin V/Propidium Iodide (PI) Assay was performed following the manufacturer's guidelines, and the cells were subsequently analyzed using flow cytometry.

### Caspase-3 activation assay

Caspase-3 activity was evaluated using the fluorescein isothiocyanate isomer (FITC) Active Caspase-3 Apoptosis Kit. In brief, cells were seeded at a density of 1 × 10^6^ cells per P10 dish and incubated overnight. Following treatment with 0.25, 1, or 2 μg/mL HMTA or DMSO for 24 h, cells were collected, fixed, and permeabilized with BD Cytofix/Cytoperm solution at 4 °C for 20 min (Becton-Dickinson, NJ, USA). Cells were subsequently incubated with a FITC-conjugated anti-active caspase-3 antibody for 30 min at room temperature. Caspase-3 activity was assessed via flow cytometry and analyzed with WinMDI 2.9 software.

### Cell cycle analysis

Pancreatic cancer cells were synchronized using a double thymidine block. The cells were cultured with 2 mM thymidine for 18 h, then switched to a thymidine-free medium for 6-8 h, and subsequently returned to a medium containing 2 mM thymidine for 12 h. After synchronization, the cells were washed twice with polybutylene succinate (PBS) and treated with either DMSO or specified concentrations of HMTA for 24 h in regular culture media. Cells were fixed overnight with 70% ethanol at 4 °C before staining with PI (Sigma-Aldrich). The staining protocol included washing the cells twice with ice-cold PBS, resuspending them in a solution containing RNase A (50 μg/mL), PI (40 μg/mL), and PBS, and incubating at 37 °C for 30 min. Cells were collected every 4 h for analysis, and flow cytometry was used to analyze the stained cells.

### Western blot analysis

In brief, cells (1 × 10^6^) were exposed to specified concentrations of HMTA or DMSO for 24 h. After treatment, cells were washed and lysed with RIPA buffer containing 1% protease inhibitor on ice for 5 min, followed by 20 s of sonication. For immunoblotting, 20 μg of protein from each sample was loaded, separated, and transferred to PVDF membranes. The membranes were blocked with 5% skim milk for 1 h at room temperature and subsequently incubated with primary antibodies against cleaved-PARP (1:1000, Cell Signaling #9542), survivin (1:1000, sc-17779), Bak (1:500, sc-832), Bax (1:500, sc-7480), Bcl-2 (1:500, sc-7382), or β-actin (1:1000, sc-47778). Thereafter, the membranes were incubated with goat anti-rabbit or anti-mouse secondary antibodies, as detailed by Tsai *et al.* 12. Target proteins were visualized using the ECL Western Blotting Detection System.

### Mitotic entry analysis

Time-lapse live cell microscopy was used to evaluate mitotic progression as described by Wang *et al.* 13. Multi-position time-lapse imaging was conducted using a Leica DMI6000 inverted microscope (Leica Microsystems Inc., Wetzlar, Germany), featuring an HCX PL FL 20x/NA0.4 objective and an Andor Luca R EMCCD camera. Differential interference contrast images were captured to observe cell morphology and mitotic progression, with imaging performed every 10 min over an 18-hour period. Metamorph software (Molecular Devices, USA) was used for image analysis and cell counts.

### Confocal immunofluorescence microscopy

Briefly, BxPC-3 and PANC-1 cells underwent a 12-hour treatment with HMTA. The cells were fixed using 4% formaldehyde and permeabilized with 0.1% Triton X-100. Following a blocking step with 1% bovine serum albumin, cells were incubated overnight at 4 °C with the primary antibody (α Tubulin (B-7): sc-5286, SANTA CRUZ, 1:800). After washing, cells were incubated with the secondary antibody (m-IgGκ BP-CFL 488: sc-516176, SANTA CRUZ, 1:200) for 1 h at room temperature. Three times of PBS washes were performed to eliminate unbound antibodies, and cells were subsequently stained with ProLong™ Gold Antifade Mountant with DAPI (Invitrogen™). The stained cells were observed using a confocal microscope (Olympus FV1000, Olympus Inc, Center Valley, PA). The ratio of cells with abnormal spindle organization was determined by counting five high-power field areas at 400× magnification.

### Molecular docking

Molecular docking calculations were performed using the AutoDock Vina program within PyRx software. The target protein, α-β tubulin (PDB ID: 1JFF), was retrieved from the RCSB Protein Data Bank based on relevant literature. The structure HMTA was drawn using ChemDraw Professional. Taxol served as the standard reference drug, and its structure was obtained from the PubChem database. The receptor for docking was α-β tubulin, with HMTA and Taxol designated as ligands. The molecular docking coordinates for HTMA were established as follows: center x = -1.1365, center y = -1.5666, center z = 3.9262 for β-tubulin; center x = 38.764, center y = -1.9068, and center z = 2.8688 for α-tubulin. Similarly, the molecular docking coordinates for the Taxol were established as follows: as center x = -1.1365, center y = -1.5666, center z = 3.9262 for β-tubulin; center x = 38.764, center y = -1.9068, and center z = 2.8688 for α-tubulin. Biovia Discovery Studio software was used to visualize the molecular interactions between ligands and receptors.

### Cell-free tubulin polymerization assay

A tubulin polymerization assay kit was used for cell-free tubulin polymerization analysis. According to the manufacturer's guidelines, the reaction mixture was prepared on ice, consisting of Buffer 1, tubulin glycerol buffer, GTP stock, and Tubulin stock, for inhibitor detection. Test compounds, paclitaxel, CaCl_2,_ or HMTA, were added to a black 96-well plate, and samples were prewarmed at 37 °C for 1 min. After pipetting 50 µL of the tubulin reaction mix into the wells, the reaction was initiated at 37 °C using a SpectraMax® iD3 multi-mode microplate reader (Molecular Devices, USA). Changes in fluorescence intensity were recorded via kinetic readings at 37 °C for >90 min (integration = 1 min). Microtubule polymerization was tracked by the increased fluorescence resulting from incorporating a fluorescent reporter into the expanding microtubules.

### BxPC-3 xenograft tumor model

The xenograft tumor assay was performed according to the method outlined by Chen *et al.* 14. Six- to eight-week-old male nude mice were used for the study. In total, 1 × 10^6^ BxPC-3 cells mixed with 50% Matrigel were injected subcutaneously into each mouse. Thereafter, mice were randomly assigned to three groups (n = 6) and received intraperitoneal injections every other day for 49 d with either the vehicle or HMTA at doses of 1 or 2 mg/kg body weight. Tumor volumes (calculated as length × width² × 0.52) and mouse weights were recorded every other day or three times a week. When the tumor volume reached 1,000 mm³, mice were euthanized and the tumors were removed, weighed, and photographed. All experimental procedures were approved by the Institutional Animal Care and Use Committee of Kaohsiung Medical University (IACUC No. 108100).

### Statistical analysis

Data is expressed as mean ± standard deviation. Significant differences between the treatment and control groups were evaluated using the Student's two-tailed t-test, and p <0.05 was considered statistically significant.

## Results

### 4-Hydroxy-2-methoxy-6-tridecylphenyl acetate reveals a potent antitumor activity in pancreatic cancer cells

**Figure 1:**
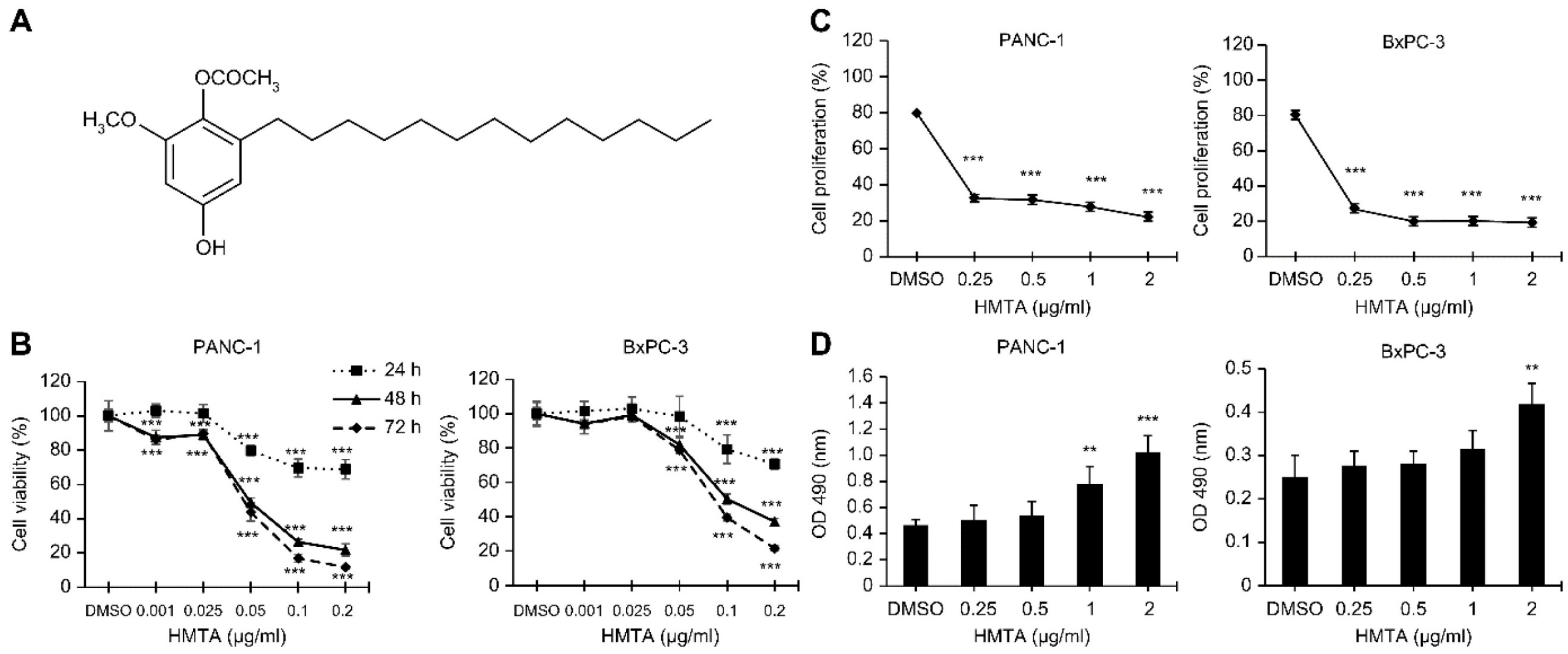
** HMTA displays cytotoxic activity against pancreatic cancer cells. (A)** Chemical formula of HMTA. **(B, C, D)** Pancreatic cancer cell lines, PANC-1 and BxPC-3, were treated with HMTA at varying concentrations for the periods indicated in (B) and 24 h in (C and D). Cell viability (B), proliferation rate (C), and cell death (D) were determined using the MTT, BrdU incorporation, and LDH assays, respectively. The data are presented as the mean ± SD of n = 3 replicates. **P <0.01, ***P <0.001 compared with vehicle-treated cells.

Initially, we validated the cytotoxic effects of HMTA on pancreatic cancer cell lines. Results from the MTT assay revealed that HMTA exhibited significant cytotoxicity towards PANC-1 and BxPC-3 cell lines (P <0.001, respectively). The IC_50_ (the concentration at which 50% of cell growth is inhibited) values of HMTA for PANC-1 and BxPC-3 cells at 48 h were 0.049 and 0.1 μg/mL, respectively. The cytotoxic effect observed in both cell lines was dose- and time-dependent, as demonstrated by the MTT assay results (Figure 1B). To evaluate the specificity of HMTA's cytotoxic effects, we also tested its impact on HPDE-6/E6E7, an immortalized human pancreatic duct epithelial cell line. The IC_50_ values for HMTA in HPDE-6/E6E7 cells were greater than the highest tested dosage (0.2 μg/mL) at 24 and 48 h, with an IC_50_ of 0.067 μg/mL at 72 h (Supplementary Figure 1). These results indicate that HMTA exhibits less cytotoxicity towards normal pancreatic cells compared to pancreatic cancer cells. The MTT assay data for HPDE-6/E6E7 cells have been included as supplementary material. Results for the BrdU incorporation assay showed that HMTA significantly inhibited proliferation of PANC-1 and BxPC-3 cells. At a concentration of 0.25 μg/mL, HMTA reduced BrdU incorporation by >50% (Figure 1C). Moreover, at higher concentrations (1-2 μg/mL), HMTA markedly increased the release of LDH in PANC-1 and BxPC-3 cell lines, indicating that HMTA treatment caused substantial cell damage (Figure 1D). These findings suggested that HMTA possesses substantial anti-pancreatic cancer cell activity.

### 4-Hydroxy-2-methoxy-6-tridecylphenyl acetate induces apoptosis in pancreatic cancer cell lines

**Figure 2:**
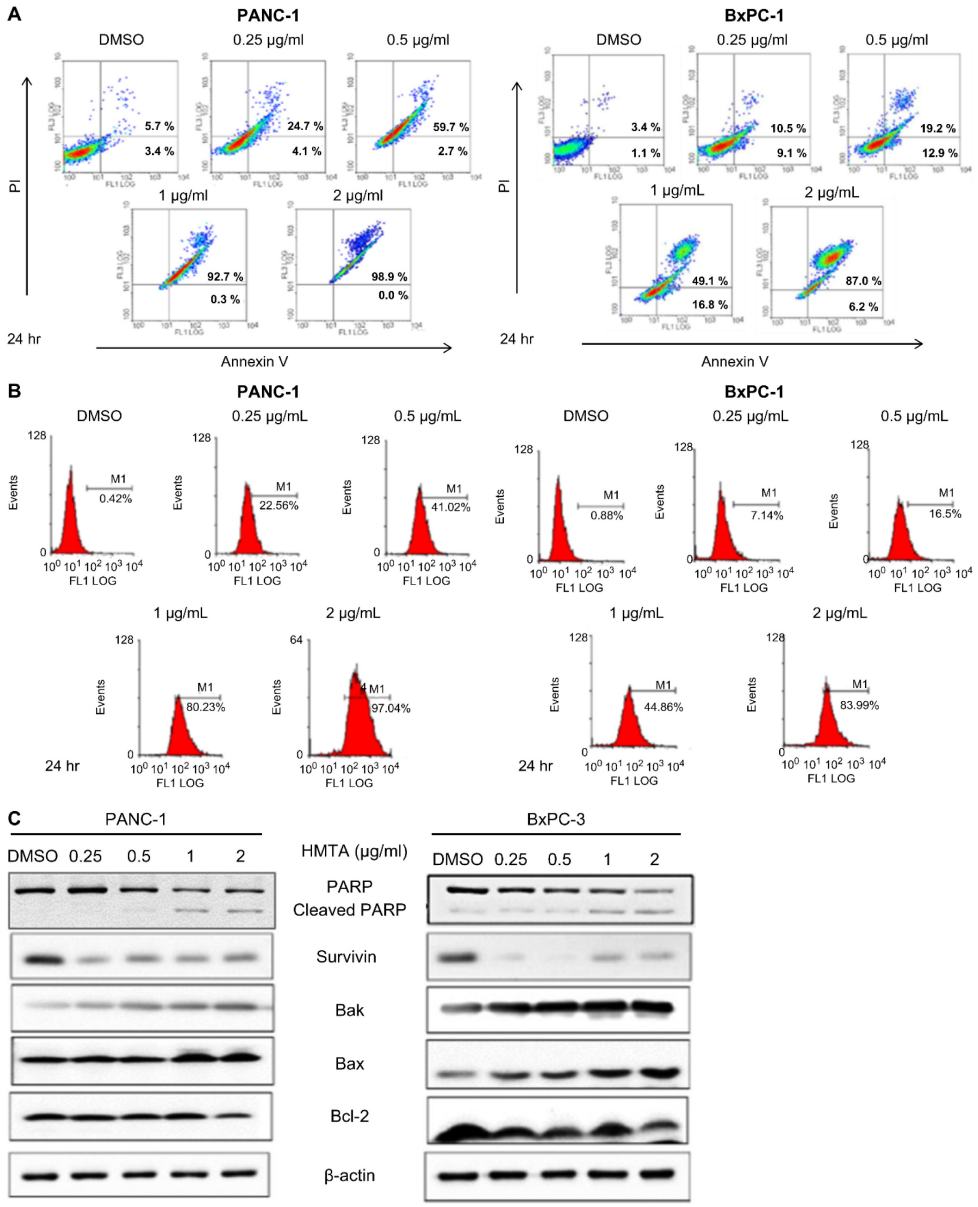
** HMTA induces apoptosis in pancreatic cancer cell lines.** Both cell lines were treated with HMTA at the indicated concentrations for 24 h, followed by Annexin V/PI staining analysis **(A)** or Active Caspase-3 Apoptosis Assays **(B)**. DMSO served as a vehicle control. **(C)** PANC-1 or BxPC-3 cells were treated with the indicated concentrations (0-2 μg/mL) of HMTA for 24 h. Whole cell lysates of PANC-1 or BxPC-3 were subjected to western blot analysis for PARP, surviving, Bak, Bax, Bcl-2, and β-actin.

We investigated whether HMTA induces apoptosis in pancreatic cancer cells. Results from the Annexin V-PI staining assay showed that HMTA at a concentration of 0.25 μg/mL increased apoptosis by approximately 20% in PANC-1 cells and approximately 15% in BxPC-3 cells. As the concentration of HMTA increased, the number of cells undergoing apoptosis was increased. At a concentration of 2 μg/mL, >90% of the cells had undergone apoptosis (Figure 2A). The results from the active caspase-3 apoptosis assays corroborated these findings. At a concentration of 0.25 μg/mL, HMTA resulted in approximately 20% and 7% caspase-3 activation in PANC-1 and BxPC-3 cells, respectively. A concentration of 2 μg/mL of HMTA induced approximately 97% and 84% caspase-3 activation in PANC-1 and BxPC-3 cells, respectively (Figure 2B). Furthermore, western blot analysis indicated that HMTA increased the expression of cleaved PARP, Bak, and Bax in these two cell lines while decreasing the expression of Bcl-2 and survivin (Figure 2C). Collectively, these results demonstrated HMTA's ability in inducing apoptosis in pancreatic cancer cells.

### 4-Hydroxy-2-methoxy-6-tridecylphenyl acetate arrested pancreatic cancer cells at the G2/M phase

**Figure 3:**
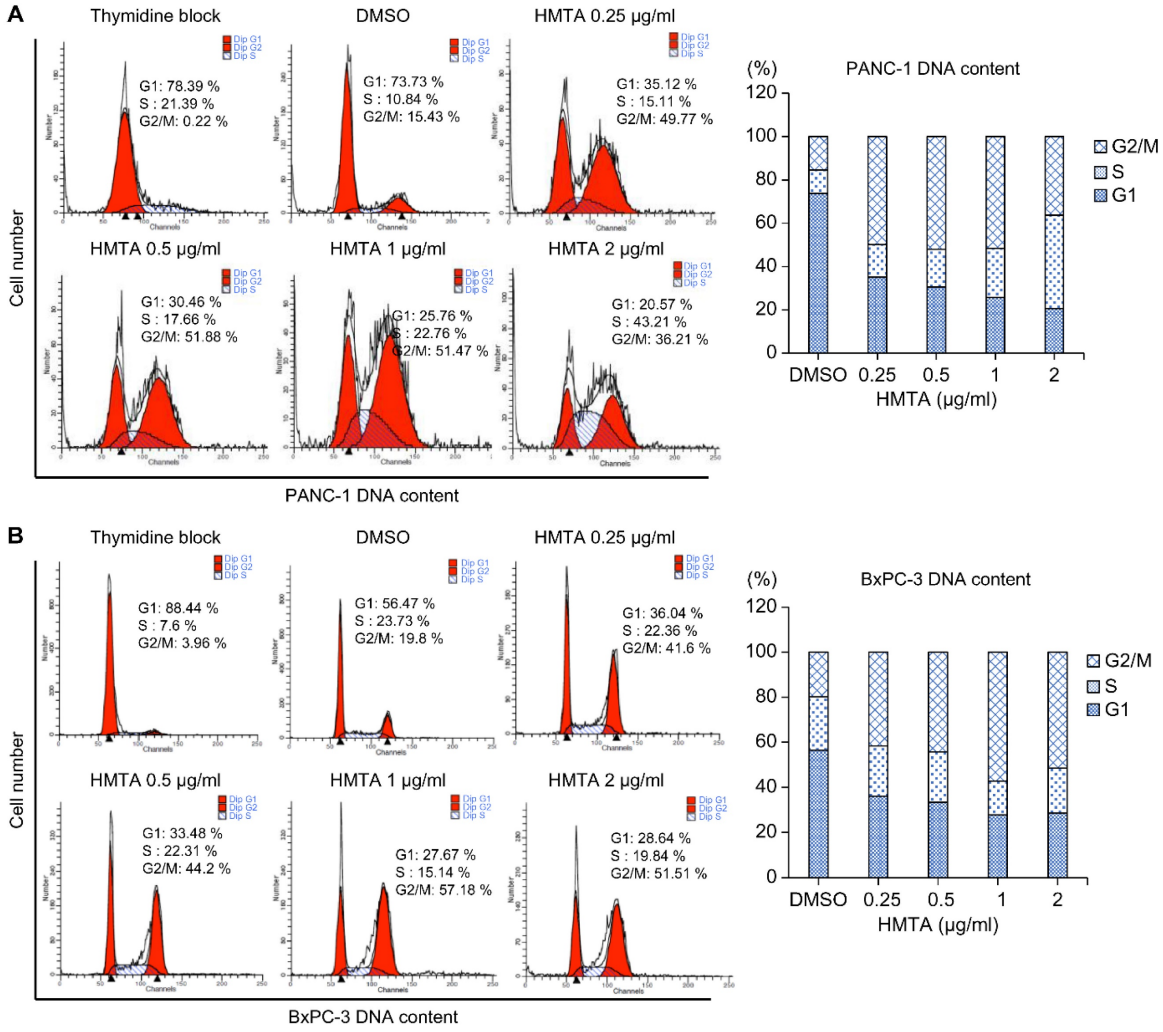
** HMTA arrested pancreatic cancer cells at the G2/M phase.** PANC-1 **(A)** and BxPC-3 **(B)** cells were exposed to different concentrations of HMTA for 24 h. Distributions of the cells in the G1, S, and G2/M stages of the cell cycle after the double thymidine block were determined via flow cytometry.

We aimed to determine whether HMTA impacts the cell cycle. To do so, we synchronized PANC-1 and BxPC-3 cells at the G1/S phase boundary using a high concentration of thymidine. After washing off the thymidine, cells were divided into groups that were treated with indicated concentrations of HMTA for 24 h, and followed by cell cycle analysis. As the concentration of HMTA increased (from 0.25 to 2 μg/mL), the proportion of G2/M cells were elevated from 15% to 36% in PANC-1 and 19% to 51% in BxPC-3 cells (Figure 3A and 3B).

### 4-Hydroxy-2-methoxy-6-tridecylphenyl acetate blocked mitotic entry and induced multinucleation

**Figure 4:**
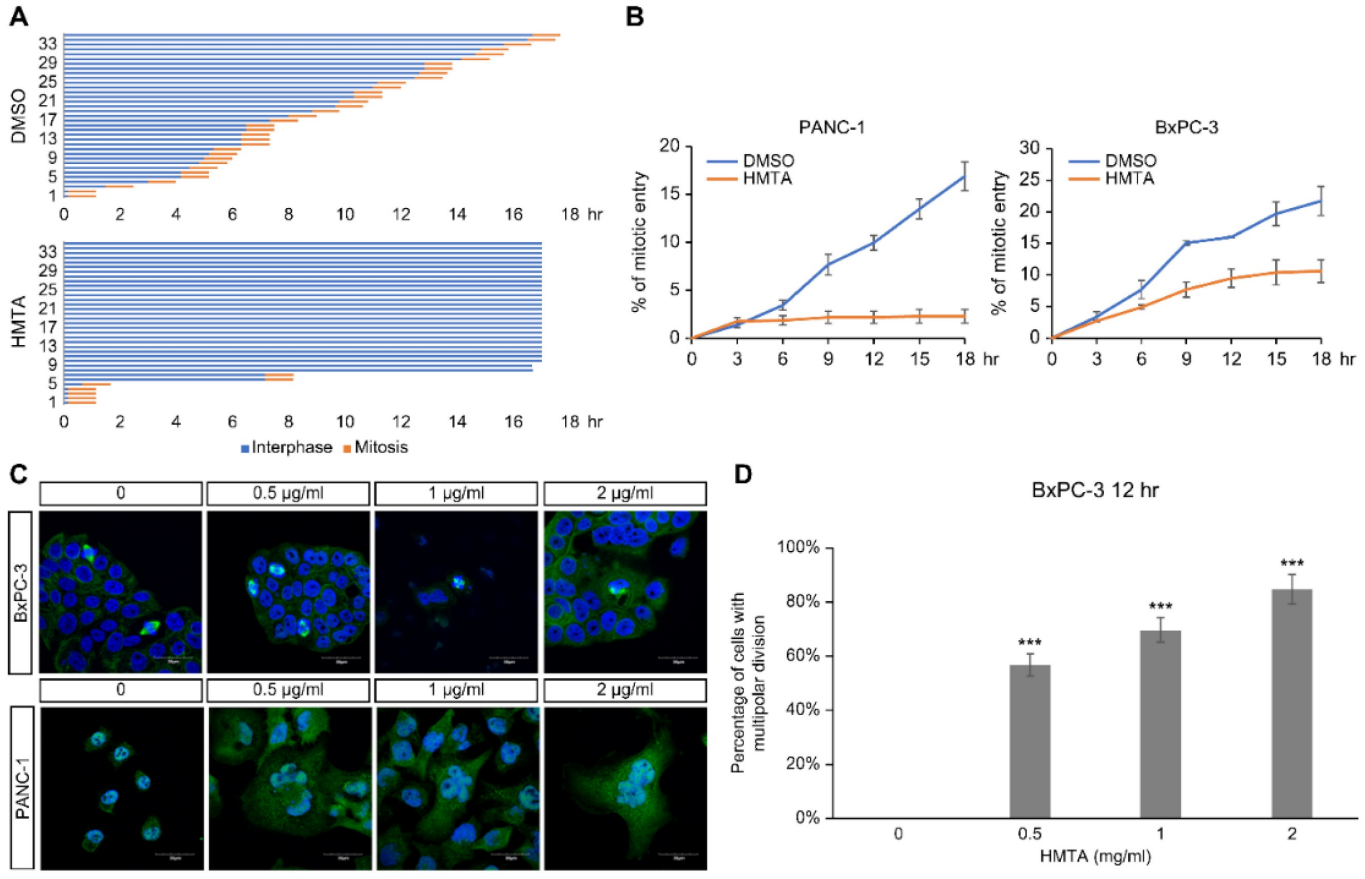
** HMTA-induced mitotic defects. (A)** Asynchronous PANC-1 cells were imaged continuously for 18 h following DMSO or 1 μg/mL HMTA treatments. Interphase cells are labeled in blue, and mitotic duration of cells is labeled in orange. Thirty-five PANC-1 cells were analyzed per condition. **(B)** Accumulative mitotic entry during live cell imaging is plotted as a function of time for PANC-1 and BxPC-3 cells. **(C)** Representative immunofluorescence images of PANC-1 and BxPC-3 cells following treatment of DMSO or HMTA at indicated concentrations for 12 h. Cells were stained with α-tubulin (green) and counterstained with DAPI. **(D)** The percentage of BxPC-3 cells with multipolar division following HMTA treatment was quantified.

To clarify the impact of HMTA on cell cycle progression, time-lapse microscopy was used to monitor cell cycle progression of individual cells over a period of 18 h. For each panel, 35 cells were recorded from the start of live imaging, and the time of mitotic entry was marked for each cell to calculate the accumulation of mitotic events and duration in mitosis over time. HMTA treatment notably reduced the mitotic entry of PANC-1 (Figure 4A). No delay in mitotic progression was detected, as indicated by the same duration in mitosis among DMSO and HMTA-treated cells (Figure 4A). The accumulation of mitotic entry for PANC-1 and BxPC-3 cells upon treatment is plotted in Figure 4B. These results indicated that HMTA severely interfered with the mitotic entry of PANC-1 and BxPC-3 cells.

Immunofluorescence imaging showed that HMTA-treated PANC-1 and BxPC-3 cells displayed enlarged nuclei and multinucleation (Figure 4C). The number of cells with multipolar division increased in PANC-1 cells following HMTA treatment (Figure 4D). These data suggested that HMTA may induce multinucleation via the induction of multipolar division.

### 4-Hydroxy-2-methoxy-6-tridecylphenyl acetate interferes with tubulin polymerization

**Figure 5:**
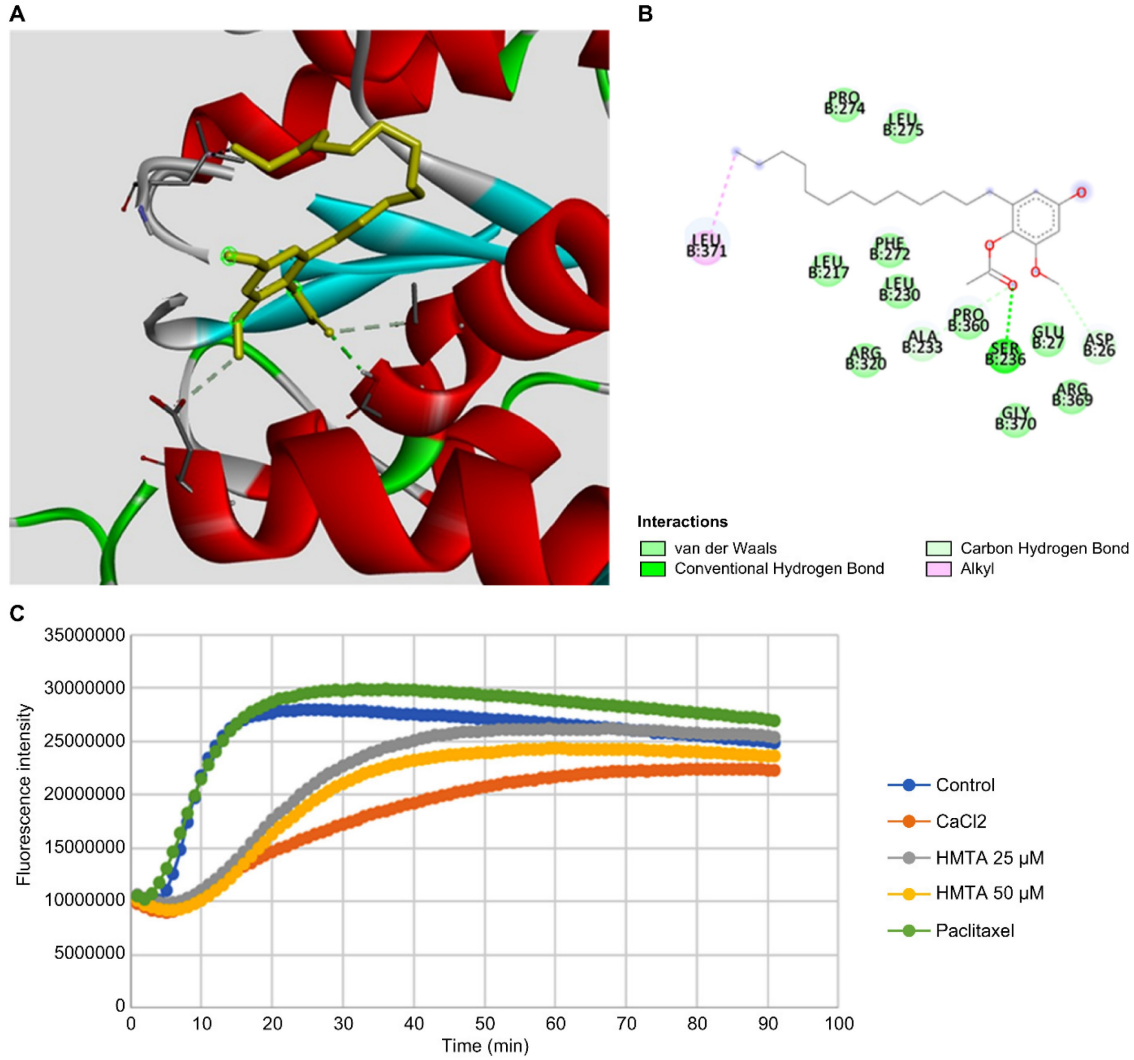
** HMTA inhibited tubulin polymerization. (A)** The proposed docking mode of HMTA in the binding site of β-tubulin (PDB code 1JFF). **(B)** The interaction illustrated by the pink line represents the hydrophobic interaction, and the green lines represent a hydrogen bond. **(C)** *In vitro* tubulin polymerization assay depicting the effects of HMTA at 25 and 50 μM, the enhancer control paclitaxel (3 μM), the inhibitor control CaCl_2_ (0.5 μM), and blank control groups. Fluorescence readings were conducted at 450 nm using the kinetic mode with excitation filters set at 360 nm, composed of 91 cycles with three readings/min.

Inspired by the observed multipolar division in BxPC-3 cells, we suggested that HMTA may affect microtubule dynamics in pancreatic cancer cells. We performed molecular docking analysis of HMTA for tubulin and identified potential interactions between HMTA and the Asp 26, Ser 236, Pro 360, and Leu 371 residues of β-tubulin (Figure 5A, B and Supplementary Table 1). Next, we examined the impact of HMTA on tubulin dynamics with an *in vitro* tubulin polymerization assay. Paclitaxel is a well-known microtubule stabilizer that speeds up polymerization and reduces depolymerization of microtubules over time (Figure 5C). As expected, CaCl_2_, a known inhibitor of microtubule polymerization, significantly delayed the speed of microtubule polymerization. Here, we demonstrated that 25 and 50 μM HMTA significantly reduced the speed of microtubule polymerization in a dose-dependent manner. Collectively, these results suggested that HMTA might function as a microtubule destabilizer by binding and delaying the polymerization of microtubules.

### 4-Hydroxy-2-methoxy-6-tridecylphenyl acetate inhibited BxPC-3 cell xenograft tumor growth *in vivo*

**Figure 6:**
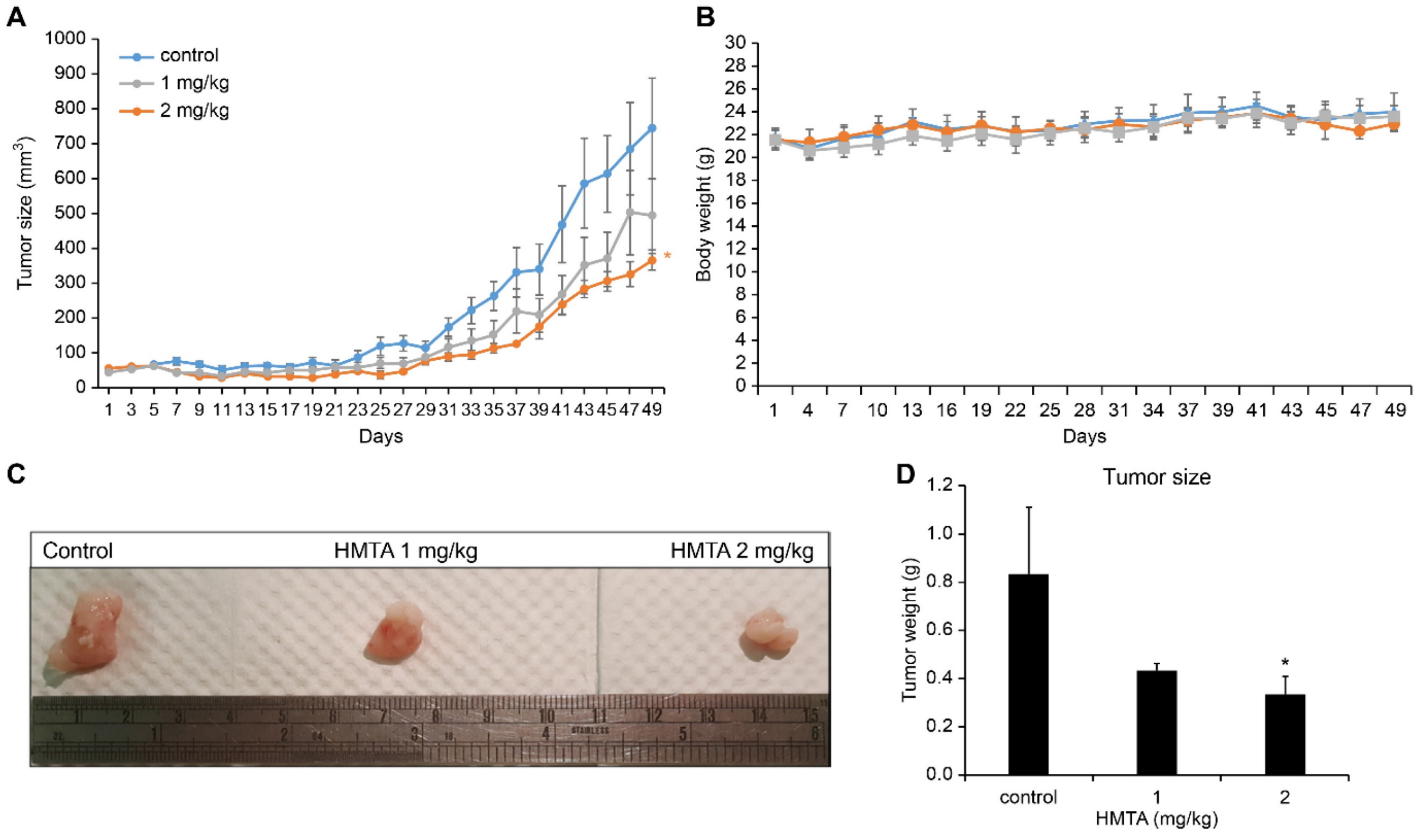
** The anti-PDAC effect of HMTA *in vivo*. (A)** The growth curve of BxPC-3 xenograft tumor in nude mice. n = 6 for each group. **(B)** The body weight of mice undergoing different treatments. **(C)** Photographs of BxPC-3 xenograft tumors. **(D)** The tumor size of all groups at day 49. Asterisk (*) indicates a significant difference from control groups (*P <0.05, t-test).

To assess the potential tumor-suppressive activity of HMTA in an *in vivo* model, we established subcutaneous xenograft tumors of BxPC-3 cells and administered varying doses of HMTA or vehicle daily via gavage. As expected, treatments of 1 and 2 mg/kg HMTA significantly reduced BxPC-3 cell xenograft tumor growth in a dose-dependent manner (Figure 6A). No noticeable impact was detected on the body weight of mice (Figure 6B). Quantitatively, administering 2 mg/kg HMTA reduced tumor size by up to 50% (Figure 6C, D).

## Discussion

Pancreatic ductal adenocarcinoma (PDAC) remains a global health challenge owing to its high mortality rate, late-stage diagnosis, and limited treatment options. Surgical intervention offers curative potential; however, a restricted fraction of patients is eligible, leading to a modest 10% five-year survival rate. Established treatments such as gemcitabine face challenges with resistance, and while the FOLFIRINOX regimen is promising, it is accompanied by considerable toxicity. This scenario highlights the urgent need for innovative therapeutic approaches. Through high-throughput screening of an indigenous plant extract library from Taiwan, we identified multiple extracts from *Ardisia virens* Kurz that demonstrated significant anti-pancreatic cancer effects. Our research on ardisiphenol D (HMTA), a potent compound from* Ardisia virens* Kurz, highlighted potential therapeutic avenues. Experimental data demonstrated that HMTA has significant cytotoxic capabilities, initiates apoptosis, and blocks mitotic processes through interfering with microtubule dynamics. Furthermore, *in vivo* experiments confirmed that HMTA markedly reduced the growth of BxPC-3 cell xenograft tumors by approximately 50%. Importantly, based on the histopathological report from BioLASCO Taiwan Co., Ltd., no treatment-related histopathological lesions were observed in the tissues of mice in both control and treatment groups. All lesions were considered spontaneous findings and unrelated to HMTA administration, underscoring its preliminary safety profile. These results suggested that HMTA could offer a new direction in PDAC treatment strategies in the foreseeable future.

The *Ardisia* genus, composed of nearly 500 species, is the largest within the Myrsinaceae family 15. Over 100 compounds have been isolated from various *Ardisia* species 16. However, only approximately 20 species have been comprehensively studied for their chemical composition and pharmacological properties 15, 16. Research specifically on *Ardisia virens* is limited. Chang *et al.* identified 14 compounds from the roots and stems of *Ardisia virens* in 2009, with seven demonstrating pronounced cytotoxicity towards MCF-7 (human breast adenocarcinoma), NCI-H460 (non-small cell lung cancer), and SF-268 (glioblastoma) cell lines, with an IC_50_ value of approximately 10 μg/mL 8. Yu *et al.* reported that ardisianone, extracted from *Ardisia virens*, exhibited anti-proliferative and apoptotic activities against hormone-refractory prostate cancer cells (PC-3 and DU-145). Notably, ardisianone inhibited the Akt and mTOR/p70S6K pathways and precipitated a rapid downregulation of survivin, activating caspase cascades 17. Lee *et al.* found that alkyl benzoquinone, derived from *Ardisia virens*, diminished glutaminase activity and glutamate levels in cells, attenuating cancer cell proliferation and inducing autophagy via AMPK and mTORC1 regulation 18. More recently, Leu *et al.* demonstrated that ardisianone, another extract from *Ardisia virens*, triggered programmed cell death in HL-60 cells via apoptosis and pyroptosis in HL-60 cells, activating caspase pathways and inducing DNA fragmentation, ultimately leading to programmed cell death 19. Our screening results align with these findings. Unlike previous research that largely focused on other cancer types, such as breast adenocarcinoma, lung cancer, and glioblastoma, this study provides the first robust evidence of the therapeutic potential of HMTA against PDAC. The discovery of HMTA's ability to inhibit tubulin polymerization, disrupt microtubule dynamics, and reduce tumor growth *in vivo* underscores its novelty. This research not only expands the pharmacological profile of *Ardisia virens* but also opens new avenues for developing targeted therapies for one of the deadliest forms of cancer.

Research on HMTA is also limited. Besides Chang *et al.*'s. findings highlighting significant cytotoxic effects on MCF-7, NCI-H460, and SF-268 cells 8, later studies by Liu *et al.* in 2011 and 2014 showed that HMTA, derived from *Ardisia brevicaulis*, inhibits the proliferation of various human cancer cells, including PANC-1 (pancreatic), A549 (lung), SGC 7901 (gastrointestinal carcinoma), MCF-7 (breast), and PC-3 (prostate) cells, via caspase-3 and caspase-9 activation and the increase of the bax/bcl-2 protein expression ratio 11. Zhu *et al.* demonstrated that HMTA from *Ardisia brevicaulis* could induce ER stress, resulting in G1 phase arrest and apoptosis in A549 cells 20. Consequently, our study focused on the anti-PDAC activity of HMTA. Consistent with prior research, we observed that HMTA possesses robust cytotoxicity against PDAC cells, enhancing Bax expression, reducing Bcl-2 and survivin expression, and activating caspase 3 and apoptosis. In a mouse xenograft model, peritoneal administration of HMTA at a dosage of 2 mg/kg every 2 d effectively reduced tumor size by over 50%. Notably, we identified that HMTA induces mitotic defects in PDAC cells, thereby resulting in cell multinucleation. Further molecular docking analysis suggests a potential interaction between HMTA and tubulin, as microtubules play a critical role in chromosome segregation during mitosis in eukaryotic cells. They are primarily regulated by polymerization kinetics 21. Targeting microtubules is a therapeutic strategy to inhibit tumor cell mitosis and in turn slow down tumor progression 22. Previous research has identified certain natural products targeting microtubule proteins, such as paclitaxel and vincristine, which are now utilized clinically 23. Our findings further suggest that HMTA combines efficacy with a favorable preliminary safety profile, warranting continued investigation as a novel microtubule-targeting agent.

## Supplementary Material

Supplementary figure and table.

## References

[1] Hu JX, Zhao CF, Chen WB, Liu QC, Li QW, Lin YY (2021). Pancreatic cancer: A review of epidemiology, trend, and risk factors. World J Gastroenterol.

[2] Rochefort P, Lardy-Cleaud A, Sarabi M, Desseigne F, Cattey-Javouhey A, de la Fouchardière C (2019). Long-Term Survivors in Metastatic Pancreatic Ductal Adenocarcinoma: A Retrospective and Matched Pair Analysis. Oncologist.

[3] Martin-Perez E, Domínguez-Muñoz JE, Botella-Romero F, Cerezo L, Matute Teresa F, Serrano T (2020). Multidisciplinary consensus statement on the clinical management of patients with pancreatic cancer. Clin Transl Oncol.

[4] Siegel RL, Miller KD, Jemal A (2020). Cancer statistics, 2020. CA Cancer J Clin.

[5] Sarvepalli D, Rashid MU, Rahman AU, Ullah W, Hussain I, Hasan B (2019). Gemcitabine: A Review of Chemoresistance in Pancreatic Cancer. Crit Rev Oncog.

[6] Mas L, Schwarz L, Bachet JB (2020). Adjuvant chemotherapy in pancreatic cancer: state of the art and future perspectives. Curr Opin Oncol.

[7] Yen CH, Chang HS, Yang TH, Wang SF, Wu HC, Chen YC (2018). High-Content Screening of a Taiwanese Indigenous Plant Extract Library Identifies Syzygium simile leaf Extract as an Inhibitor of Fatty Acid Uptake. Int J Mol Sci.

[8] Chang HS, Lin YJ, Lee SJ, Yang CW, Lin WY, Tsai IL (2009). Cytotoxic alkyl benzoquinones and alkyl phenols from Ardisia virens. Phytochemistry.

[9] Bao L, Wang M, Zhao F, Zhao Y, Liu H (2010). Two new resorcinol derivatives with strong cytotoxicity from the roots of Ardisia brevicaulis Diels. Chem Biodivers.

[10] Chen LP, Zhao F, Wang Y, Zhao LL, Li QP, Liu HW (2011). Antitumor effect of resorcinol derivatives from the roots of Ardisia brevicaulis by inducing apoptosis. J Asian Nat Prod Res.

[11] Zhao F, Hu Y, Chong C, Lu M, Chen L, Kan W (2014). Ardisiphenol D, a resorcinol derivative identified from Ardisia brevicaulis, exerts antitumor effect through inducing apoptosis in human non-small-cell lung cancer A549 cells. Pharm Biol.

[12] Tsai WC, Wang WH, Huang BC, Huang CY, Sheu JH (2021). 5-epi-Sinuleptolide from Soft Corals of the Genus Sinularia Exerts Cytotoxic Effects on Pancreatic Cancer Cell Lines via the Inhibition of JAK2/STAT3, AKT, and ERK Activity. Molecules.

[13] Wang LH, Yen CJ, Li TN, Elowe S, Wang WC, Wang LH (2015). Sgo1 is a potential therapeutic target for hepatocellular carcinoma. Oncotarget.

[14] Chen YJ, Wang WH, Wu WY, Hsu CC, Wei LR, Wang SF (2017). Novel histone deacetylase inhibitor AR-42 exhibits antitumor activity in pancreatic cancer cells by affecting multiple biochemical pathways. PLoS One.

[15] de Mejía EG, Ramírez-Mares MV (2011). Ardisia: health-promoting properties and toxicity of phytochemicals and extracts. Toxicol Mech Methods.

[16] Liu B, Liu R, Liu Q, Ashby CR Jr, Zhang H, Chen ZS (2022). The ethnomedicinal and functional uses, phytochemical and pharmacology of compounds from Ardisia species: An updated review. Med Res Rev.

[17] Yu CC, Wu PJ, Hsu JL, Ho YF, Hsu LC, Chang YJ (2013). Ardisianone, a natural benzoquinone, efficiently induces apoptosis in human hormone-refractory prostate cancers through mitochondrial damage stress and survivin downregulation. Prostate.

[18] Lee YZ, Yang CW, Chang HY, Hsu HY, Chen IS, Chang HS (2014). Discovery of selective inhibitors of Glutaminase-2, which inhibit mTORC1, activate autophagy and inhibit proliferation in cancer cells. Oncotarget.

[19] Leu WJ, Chang HS, Chen IS, Guh JH, Chan SH (2021). Antileukemic Natural Product Induced Both Apoptotic and Pyroptotic Programmed Cell Death and Differentiation Effect. Int J Mol Sci.

[20] Zhu GY, Wong BC, Lu A, Bian ZX, Zhang G, Chen HB (2012). Alkylphenols from the roots of Ardisia brevicaulis induce G1 arrest and apoptosis through endoplasmic reticulum stress pathway in human non-small-cell lung cancer cells. Chem Pharm Bull (Tokyo).

[21] Gudimchuk NB, McIntosh JR (2021). Regulation of microtubule dynamics, mechanics and function through the growing tip. Nat Rev Mol Cell Biol.

[22] Jordan MA, Wilson L (2004). Microtubules as a target for anticancer drugs. Nat Rev Cancer.

[23] Huang M, Liu C, Shao Y, Zhou S, Hu G, Yin S (2022). Anti-tumor pharmacology of natural products targeting mitosis. Cancer Biol Med.

