# Supplementary material for: Taming Pancreatic Cancer: Ardisia virens Kurz-Derived 4-Hydroxy-2-Methoxy-6-Tridecylphenyl Acetate as a Potent Tubulin Polymerization Inhibitor for Targeted Pancreatic Ductal Adenocarcinoma Therapy
Source: Int J Med Sci. 2025 Jan 13;22(3):651–61. doi: 10.7150/ijms.104112 (PMC11783067; doi:10.7150/ijms.104112)

Supplementary materials

Supplementary Table 1. Binding energy (Kcal/mol) of docking ligands HMTA and Taxol to α-tubulin and β-tubulin receptors.

Compounds	Binding affinity (Kcal/mol)	
	α-tubulin	β-tubulin
HMTA	-5.4	-5.9
Taxol	-7.6	-9.3

HMTA, 4-hydroxy-2-methoxy-6-tridecylphenyl acetate.

Supplementary Figure 1.

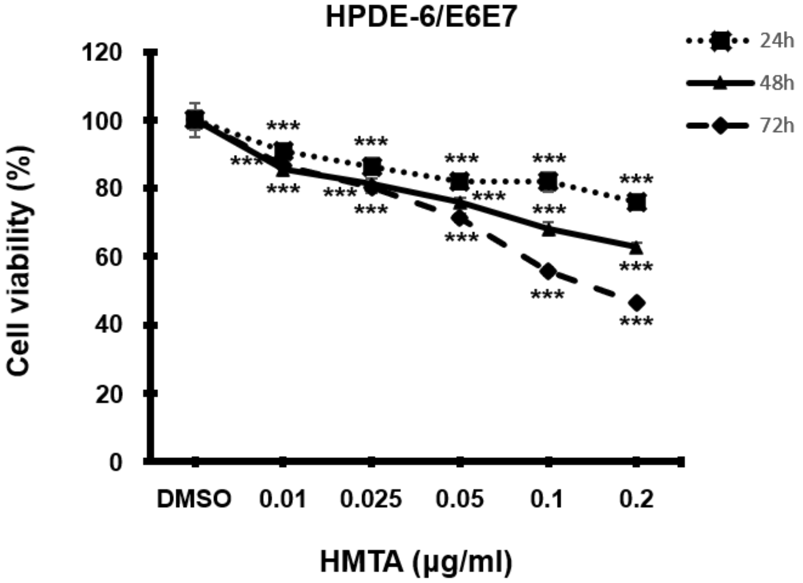

Supplement: Supplementary file 1 — Supplementary figure and table. [file ijmsv22p0651s1.pdf]
